# Women have substantial advantage in STEM faculty hiring, except when competing against more-accomplished men

**DOI:** 10.3389/fpsyg.2015.01532

**Published:** 2015-10-20

**Authors:** Stephen J. Ceci, Wendy M. Williams

**Affiliations:** Department of Human Development, Cornell UniversityIthaca, NY, USA

**Keywords:** affirmative action, women in science, bias, sexism, academic hiring

## Abstract

Audits of tenure-track hiring reveal faculty prefer to hire female applicants over males. However, audit data do not control for applicant quality, allowing some to argue women are hired at higher rates because they are more qualified. To test this, Williams and Ceci (2015) conducted an experiment demonstrating a preference for hiring women over identically-qualified men. While their findings are consistent with audits, they raise the specter that faculty may prefer women over even more-qualified men, a claim made recently. We evaluated this claim in the present study: 158 faculty ranked two men and one woman for a tenure-track-assistant professorship, and 94 faculty ranked two women and one man. In the former condition, the female applicant was slightly weaker than her two male competitors, although still strong; in the other condition the male applicant was slightly weaker than his two female competitors, although still strong. Faculty of both genders and in all fields preferred the more-qualified men over the slightly-less-qualified women, and they also preferred the stronger women over the slightly-less-qualified man. This suggests that preference for women among identically-qualified applicants found in experimental studies and in audits does not extend to women whose credentials are even slightly weaker than male counterparts. Thus these data give no support to the twin claims that weaker males are chosen over stronger females or weaker females are hired over stronger males.

## Introduction

Much has been written about the campaign to diversify faculty at American colleges and universities, an effort that started in earnest during the 1980s and continues unabated. To this end, hundreds of analyses of faculty hiring for tenure-track positions have been reported, and the temporal changes in the fraction of female and minority applicants in the American professoriate have been charted (e.g., Smith et al., 2004; Kang and Banaji, 2006; Turner et al., 2008; Niederle et al., 2013). Despite substantial gains in diversity of faculty, the dominant view appears to be that racial and gender preferences continue to be needed to counter not just historical prejudice but also current biases held by faculty—most of which may be implicit, and which result in barriers against hiring women and minorities. It is alleged that such biases create, in the words of Kang and Banaji, “threats to fair treatment—threats that lie in every mind” and that affirmative-action hiring programs should be continued until data are available to indicate such threats are over: “such data should be a crucial guide to ending affirmative action” (Kang and Banaji, 2006, p. 1063). With some notable exceptions demonstrating female-friendly hiring preferences by faculty (Williams and Ceci, 2015), there continues to be evidence of implicit and occasionally explicit biases directed at women and ethnic minorities. Although few of these demonstrations of bias concern hiring of academic science faculty, some of them are indirectly relevant. The present experiment was undertaken to determine whether gender differences trump applicant quality in tenure-track hiring decisions.

### Stereotypes, hiring bias, and gender congruity

A growing literature reveals people are apt to explicitly associate science with men, including not only students but also scientists (Smyth and Nosek, 2015), and that such stereotypes are pervasive, as shown recently by Miller et al. (2015). In their transnational analysis, Miller et al. showed higher female enrollment in post-secondary course-taking in nations with weaker implicit and explicit gendered stereotypes regarding science. Such stereotypes can lead to biased evaluations against women in so-called gender-incongruous contexts, such as in STEM fields in which men have historically been dominant (engineering, physics, economics, computer science, geosciences, and mathematics). This form of bias is particularly likely to emerge when information about applicants' competence is unavailable or when the evaluators are not experienced professionals. For example, Ernesto Reuben and his colleagues (Reuben et al., 2014) asked nearly 150 men and women (mostly undergraduates) to add a string of four 2-digit numbers. They were given 4 min to do as many additions as possible. The authors then assigned the role of hiring manager to nearly 200 male and female students, who were asked to decide whom among these 150 students to hire. Afterward these managers were given an implicit bias test. The authors found that men were hired at twice the rate of women; most of the students playing the role of hiring manager believed men were better at math and science. Even when informed of superior arithmetic scores by women, some hypothetical managers continued to prefer to hire men. In their “cheap talk” condition (which had the largest gender bias), applicants selected the lower performing male over the higher performing female in 29% of the cases compared to selecting the lower performing female over the higher performing female in only 2% of the cases. Hence, in that study, the pro-male bias trumped even applicant quality. Taken together, these transnational and experimental studies indicate that implicit biases and sometimes explicit ones can lead to fewer women preparing for a career in STEM and ultimately being hired.

Studies of gender biases suggest that stereotypes are not always activated but rather are invoked when information about applicants is limited or ambiguous or when evaluators lack motivation to be careful. In such situations stereotypes can reduce cognitive load during decision-making. However, relying upon stereotypes may be unnecessary when information about applicants indicates unambiguously high competence, as in the case with tenure-track hiring. In their recent metaanalysis, Amanda Koch and her associates found that gender-role congruity bias was largest when so-called “individuating information” that was informative of applicants' competence was ambiguous or not clearly diagnostic of success. They reported that sex bias shrinks in male-dominated fields when diagnostic information about applicants' competence is available (see Koch et al., 2015, pp. 130–131). The authors reported near-zero bias when female applicants were evaluated by experienced professionals in male-dominated fields if information regarding their competence was available (*d* = 0.02). This finding is relevant to the Reuben et al. hiring manager study because the evaluation was not done by experienced professionals, and in one version student managers were significantly less biased when they were supplied with the women's actual arithmetic scores, albeit some smaller number of student managers still exhibited a male hiring bias even in this condition. Typically, however, the studies in the metaanalysis examined applicants with equally strong records, thus telling us little about whether bias occurs for female applicants possessing inferior credentials, as some have alleged (e.g., Niederle et al., 2013). We directly address this lacuna in the current experiment.

Relatedly, Moss-Racusin et al. (2012) found that both male and female faculty preferred to hire, remunerate, and mentor a male applicant for a lab manager post than an identically-qualified female applicant. However, the lab manager post was baccalaureate-level and the lab manager applicant was depicted as ambiguously-competent rather than as unambiguously stellar. Thus, the Reuben et al. and Moss-Racusin et al. experiments leave unanswered the question of whether such bias would be found in hiring applicants for professorships under conditions in which experienced faculty have motivation to be careful and possess diagnostic information about applicants' competence—in other words, the real-world conditions under which faculty are usually hired.

### Background for the present study

In the present study we report findings from an ongoing program of experimental research aimed at examining biases in the hiring of women scientists in male-dominated fields in the academy. The major focal question in the current study is: How much do gender-related biases trump preferences for the candidate with the highest quantitative competence index, based on publications, letters, interview, and job talk? Recent experimental evidence indicates that when evaluators are themselves experienced professionals, women applicants for professorships are preferred over equally-competent men when both are depicted realistically, as identically and unambiguously stellar (Williams and Ceci, 2015). Here we ask whether this preference for female applicants will extend to situations in which women are quantitatively slightly weaker than men.

Many blue-ribbon panels and national organizations argue for the continued use of preferential hiring programs because biased hiring is viewed as a cause of women's underrepresentation in academic science, by “inadvertently foreclosing consideration of the best-qualified persons by untested presuppositions which operate to exclude women and minorities” (AAUP, 2014). Notwithstanding the recent pro-female hiring data of Williams and Ceci, there are recent empirical data implying that hiring is sexist and that it possibly forecloses the prospects of the best-qualified female applicants. However, none of these data concern the hiring of academic science faculty by professionals who possess diagnostic information, but they nevertheless are relevant. Below we describe a survey study and an experiment that are relevant to gender bias in academic hiring, even though neither actually involves hiring of professors in male-dominated fields.

Sheltzer and Smith (2014) surveyed biology department web pages and departmental directories to ascertain the numbers of graduate students and postdoctoral researchers employed by faculty members. They found that elite male faculty (winners of lifetime awards, members of the National Academy of Sciences, recipients of funding by the Howard Hughes Medical Institute) employed fewer female graduate students and postdoctoral researchers than did elite female faculty, who did not exhibit a gender asymmetry. New assistant professors in biology were disproportionately comprised of individuals who came from these elite laboratories, which had an overabundance of male grad students and postdocs, thus reflecting a seeming causal loop. However, two features of this study merit mention: first, biology is a field in which women are well represented among both PhD recipients and among the professoriate, so it is unlikely to be the ideal field in which to detect gender bias. Second, because this was not an experiment, it leaves open alternative explanations for the observed gender asymmetry, such as whether female postdocs self-selected (i.e., were more likely to apply to work with female faculty). Despite these concerns, the findings are suggestive of a male faculty bias in recruiting and appointing postdocs that can eventuate in more male professors being hired, despite the fact that biology is a field that appears to be female-friendly.

There is one experiment in the last 30 years that has addressed the question of sex bias in the hiring of professors; it was conducted by Steinpreiss and her associates 16 years ago (Steinpreis et al., 1999). They found faculty of both genders preferred to hire the male applicant over the identically-qualified female applicant. However, there are two features of this experiment that limit its applicability: first, it examined bias in only one field, psychology, which is the field in which women are best represented—psychology has the largest fraction of women professors of all STEM fields, constituting the majority of faculty. Second, Steinpreiss et al. did not find a preference for hiring a man over a woman when the hypothetical applicants were depicted as unambiguously stellar senior faculty applicants (considered for early tenure). The reason these points are noteworthy is that Koch et al.'s metaanalysis found small-to-moderate sex bias in male-dominated jobs when applicants had average or ambiguous competence (*d* = 0.29) but, as noted above, no bias when applicants had high competence (*d* = 0.02) or when evaluators were motivated to be careful (*d* = 0.01), both conditions that characterize tenure-track hiring. For hiring tenure-track professors in male-dominated fields such as engineering, physics, and economics, experienced professionals might be expected to exhibit little or no sex bias when evaluating applicants who are unambiguously competent. Finally, some evidence suggests that an implicit stereotypic association of race with violence in a videogame simulation did not lead to racist behavior when participants held relatively high implicit negative attitudes toward prejudice (Glaser and Knowles, 2008). This suggests that motivation against possessing or demonstrating bias influences behavior and attitudes of even those possessing implicit biases.

In contrast to experiments showing hiring bias, Williams and Ceci (2015) reviewed 8 large-scale audits of actual hiring that indicate women are preferred for tenure-track hiring in the real world. For example in a large National Research Council (NRC) (2009) analysis, women were hired at rates higher than their application numbers in every field assessed at the 89 research universities the NRC panel studied: in mathematics, women constituted 20% of applicants but 32% of hires; in electrical engineering women were 11% of applicants but 32% of hires; in chemistry women were 18% of applicants but 29% of hires; and in physics they were 12% of applicants but 20% of hires. Similar pro-female hiring data were reported in the National Computer Research Association hiring report for professorships in computer science: “as new PhDs, women submitted far fewer applications than men but received many more offers per application. Female new hires applied for only 6 positions (compared with 25 for men), obtained 0.77 interviews per application (vs. 0.37 for men), and received 0.55 offers per application (vs. 0.19 for men). Obviously women were much more selective in where they applied, and also much more successful in the application process.” Against this backdrop of actual hiring data showing a preference for female applicants, a goal of this program of research has been to determine whether this hiring advantage occurs because women applicants are more qualified than men. Williams and Ceci (2015) showed in their experiments this is not what is driving the female hiring preference because women applicants continue to be preferred over male applicants who are equally qualified. This is in contrast to frequent claims to the contrary.

### Present study

In a recent series of experiments, Williams and Ceci (2015) asked a nationally stratified sample of 873 faculty from four academic fields (economics, psychology, biology, and engineering) to rank two otherwise identically-qualified hypothetical finalists for a tenure-track assistant professorship in their department. These identically-qualified finalists were referred to as Dr. X and Dr. Z and they were presented to faculty with identical quantitative ratings of their candidacy based on their research, job talk, letters, and interview; the sole difference between them was their gender. Faculty were informed that Dr. X and Dr. Z were both rated 9.5 by their departmental colleagues on the basis of their publications, interview, letters, and meetings, where 10.0 = outstanding/exceptional and 1 = cannot support for tenure-track hiring. Thus, Drs. X and Z were depicted as unambiguously strong applicants, which is realistic for tenure-track applicants who have made it to the short list of finalists in searches that often generate hundreds of PhD applicants.^1^ Faculty preferred to hire the female 2-to-1 over her identically-qualified male counterpart. This strong pro-female bias was found in all four fields and by faculty of both genders with the exception of male economists who showed no preference between equivalently-qualified female and male applicants. Because of its stratified national sampling and use of sampling weights, the Williams and Ceci (2015) findings were representative of the size of the ratio at all types of institutions, from small teaching-intensive colleges to large, research-intensive ones.

There were two features in Williams and Ceci's experimental design that were implemented to obscure the true purpose of their experiment, one of which is relevant in the present context. To obscure the true nature of their hypothesis so that faculty would not realize they were being assessed to determine whether they harbored sexist biases in hiring, Williams and Ceci disguised the study to appear as a competition between different personalities. In actuality the personalities were counterbalanced with gender and varied in a between-subjects design. In addition to the use of this personality disguise, there was another ploy used to minimize faculty respondents' awareness; it was the addition of a third applicant, a foil. In addition to pitting an equally-qualified Dr. X against a Dr. Z, Williams and Ceci added a third short-listed competitor who was pretested to be slightly inferior to X and Z, labeled Dr. Y. Unlike Drs. X and Z who were both given quantitative scores of 9.5, Dr. Y was given 9.3, which although still very strong is slightly inferior. In the Methods section we describe this feature in more detail because it is a central aspect of the present study. Thus, the inclusion of these two features—a slightly lower-rated foil (Dr. Y) and the counterbalanced adjectives—served to disguise the true purpose of the experiment. And the misdirection appeared to work: A survey of 30 faculty in their study reported no suspicion that the experiment had to do with gender preference in hiring.

Summing across numerous analyses, Williams and Ceci reported the odds of preferring a woman over an identically-qualified man was roughly 2-to-1. Importantly for the purpose of the present experiment, only 2.53% of faculty preferred to hire Dr. Y over his slightly stronger competitors, Drs. X and Z. In a subsequent experiment that excluded the Dr. Y foil, these researchers asked faculty to rate only one applicant (either a female or male finalist), to avoid implicit competition between a woman and man. Faculty assigned their own quantitative scores to the applicant they were sent to evaluate. Again, there was a preference for women, with faculty of both genders giving the female candidate a higher quantitative score than other faculty gave the identically-qualified male candidate. This latter finding suggests that faculty have internalized the norm of gender diversity and were not merely responding in a manner that is politically correct or to exhibit some other form of impression-management, because faculty had no knowledge that other faculty were evaluating the identical accomplishments in the form of an opposite-sex applicant.

These results raise an intriguing question regarding the pervasiveness of the preference for women: Would it still be observed if the Dr. Y foil was a woman instead of a man? If Dr. Y was a slightly less accomplished female finalist as compared to the two male finalists, would faculty still reject her—that is, would they still choose her only 2.53% of the time as was found when Dr. Y was a male? Or would the desire for gender diversity among faculty be sufficiently strong that they would prefer to hire a slightly less accomplished female Dr. Y over more accomplished male applicants? This is the question we attempt to answer in the current experiment. It will shed light on the extent of faculty's desire to diversify the academy: It is one thing to find that faculty of both genders prefer to hire a female applicant over her identically-qualified male counterpart by a ratio of 2-to-1, but it is another matter to ask whether this preference for female applicants extends to a preference to hire a slightly weaker female applicant, one described as 9.3 on a 10-point scale who is competing against two males who are described as 9.5.

Thus, the current experiment consisted of a comparison of a woman assigned a slightly lower quantitative score competing against two men assigned a slightly higher quantitative score, all of whom were competing for the same assistant professorship. We used the same 9.3 vs. 9.5 quantitative scores used by Williams and Ceci (2015) because their survey provides a national base-rate for faculty expectations for this contrast. If a preference is found for a female finalist depicted as 9.3 over men depicted 0.2 points higher, then subsequent contrasts between even lower-rated females would be in order. But first we sought evidence of preferential hiring of women who are only slightly weaker than their male competitors.

## Methods

### Participants

The pool of potential faculty participants was assembled by drawing a national stratified sample of 694 tenured/tenure-track professors (half female, across all ranks). This was done by randomly sampling from online directories for Carnegie Foundation's 3 Basic Classifications of: (a) Doctoral (combining all three levels of doctoral intensity), (b) Master's institutions (combining all three levels—small, medium, and large), and (c) Baccalaureate institutions (combining all three levels of such institutions). This sample of 694 professors was drawn equally from four popular fields, two math-intensive ones in which women faculty are greatly underrepresented— < 15% (engineering, economics)—and two non-math-intensive fields (biology, psychology) in which women faculty are well represented and are considered to have achieved what gender equity advocates regard as a critical mass, although even these fields still produce significantly more female PhDs than the female fraction of total professorships. There were two constraints in randomization. One was that for an institution to be included it had to have programs in at least three of the four fields. This was true of all doctoral institutions in the sampling frame, but it excluded many small colleges that lacked two or more of the four fields, and over half of the nation's combined master's programs. The second constraint was that only tenured or tenure-track faculty were included in the sample frame; off-line faculty (emeriti, adjuncts, lecturers, instructors, courtesy faculty members, and visiting professors) were excluded, as only faculty who actually vote on tenure-track hiring were desired as subjects.

Overall, out of the 694 faculty who were assigned to one of two conditions, 252 responded with full data (36.3%): 158 rated a male Dr. X who was pitted against a female Dr. Y and a male Dr. Z; and 94 rated a female Dr. X who was pitted against a male Dr. Y and a female Dr. Z.

### Materials

Two sets of materials were used, the first containing profiles of two *male* applicants, Dr. X and Dr. Z, with identical scholarly qualitative scores but differing in gendered adjective descriptors (“kind, socially-skilled, creative” vs. “analytical, competitive, powerhouse”). As noted, these descriptors were used to disguise the actual hypothesis, leading raters to believe the research question was whether they preferred one type of individual over the other. These gendered descriptors were counterbalanced so that half the faculty received Dr. X portrayed as a male “analytical, competitive, powerhouse” competing against Dr. Z as a male “kind, socially-skilled, creative” colleague, and half received Dr. X and Z portrayed with the opposite terms. Dr. Y was described as “shy and reserved,” which is more negative than “socially skilled” or a “real powerhouse,” and in the chair's notes some concern was raised about Y's teaching performance, whereas no concern was raised for X or Z. Thus, the quantitative “pre-rankings” gave an explicit cue that Drs. X and Z were stronger than Dr. Y, albeit only slightly so (see Supplementary Material for one set of these materials). These different personae were the same as those used by Williams and Ceci (2015) and were based on gender congruity norms (Diekman and Eagley, 2000; Cuddy et al., 2004). Notwithstanding this systematic variation of these descriptors between faculty raters, Drs. X and Z were otherwise identical: both were rated 9.5 out of 10.0 in quality on the basis of their scholarly accomplishments, job talk, and faculty meetings. This corresponded to “impressive.”

In every contest between the male Drs. X and Z, a third candidate was added, a female Dr. Y, who was depicted as slightly lower in scholarly quality (9.3) than the male Drs. X and Z, and who was pretested with an independent group of faculty who did not participate in this experiment to ensure that raters perceived her quality as slightly lower. Dr. Y was always depicted in the same terms used by Williams and Ceci for their Dr. Y foil when he was a male, since it was established that under these conditions their male Dr. Y was chosen by only 2.53% of faculty in their large stratified national sample.

The second set of materials simply reversed the genders so that Drs. X and Z were depicted as women and Dr. Y as a man; everything else was identical.

### Procedure

Thus, the contest presented to every faculty member was to choose between three finalists for a tenure-track position, in one condition with Drs. X and Z both being male candidates of equivalent quality (9.5) and Dr. Y being a slightly lower quality female candidate (9.3), and in the other condition with these genders reversed (see Supplementary Material for materials). Faculty members were sent personal emails containing one of the counterbalanced depictions, and were asked to rank these three finalists in order of their hiring preference: first, second, and third for a tenure-track assistant professorship in their own department. The question of interest is whether faculty exhibit preferential hiring for female applicants possessing slightly lower quantitative scores than their male counterparts.

## Results

The main analysis examined which candidate was ranked first by faculty of each gender and at each type of college/university, and in each of four academic disciplines. In addition to the four disciplines (engineering, economics, psychology, biology) there were three types of colleges/universities based on the Carnegie classification (1 = doctoral, 2 = bachelors/masters, 3 = baccalaureate).

The response rates for every cell (university Carnegie type by discipline by gender, 3 × 4 × 2) were evaluated in a logistic regression. Response rates for the 252 faculty across these 24 cells were unrelated to the findings. These data were analyzed with both unweighted and weighted logistic regression models to provide a stronger test on their representativeness. Here we report only the traditional unweighted analyses but the weighted results (weighted to account for differences in the numbers of men and women in the population and in the sampling frame) were highly similar, with no result changing.

Across the 158 contests between the equivalently strong male Drs. X and Z, only 7 faculty respondents preferred the slightly weaker female Dr. Y, and one faculty rater gave tied ranks for X and Y for first place. This resulted in an overall female Dr. Y-preference of 4.8%. In the condition in which 92 faculty were asked to choose between two slightly more accomplished women—Drs. X and Z—and a slightly less accomplished male Dr. Y, only 1 out of 92 respondents chose the latter (1.2%). There was no statistical difference between Y foils when depicted as male vs. female, chi square 2.136, *p* = 0.144 (The 95% CIs for the ratio of choosing Dr. Y 7 times out of 158 contests is between 2 and 9 percent, and the ratio of choosing Dr. Y 1 time out of 92 contests is between 0 and 6 percent; the CI of the difference in proportions covers 0, ranging from −1.59 to 7.3 percent).^2^ There were no differences between the four disciplines in this male vs. female Y-preference, nor were there any differences between the three types of Carnegie institutions or between male and female faculty members, all *p* > 0.20. Finally, faculty gender did not interact with the gender of the Y foil. Basically, everyone preferred the more accomplished X and Z candidates over the less accomplished Y candidate, regardless of Y's gender. And this extended even to fields in which women are very underrepresented (engineering and economics).

In view of this finding, there was no justification for conducting a follow-up experiment in which the female Y foil was depicted as less qualified than the 9.3 value used in this experiment, given that she was not preferred even at this slightly lower level, meaning that she would not be ranked higher if she were depicted as lower in quality than 9.3 out of 10.

## Discussion

When, as in the present experiment, women candidates are depicted as slightly less accomplished than their male counterparts, they did not have a significant gender advantage in hiring, and were bypassed in favor of slightly superior male candidates 95.2% of the time, which is not significantly different from the 97.47% bypass rate of males depicted as slightly less accomplished (2.53% choosing male Y foil who had a 9.3 score) in Williams and Ceci's (2015) experiment. That is, this result is also similar to the situation in which Dr. Y is depicted as a less accomplished male competing against two stronger female Drs. X and Z. In this latter contest, the male Dr. Y is chosen only 1.2% of the time (all *p* > 0.10, n.s.). Even taking into account low power to detect differences between magnitudes this small, the hundreds of faculty in the Williams and Ceci (2015) study and the hundreds in the present study suggest that it is rare (<5%) to prefer any applicant who is depicted as even slightly weaker than her or his competitors. Apparently, academic faculty view quality as the most important determinant of hiring rankings, which suggests that when women scientists are hired in the academy it is because they are viewed as being equal or superior to male competitors.

Hence, the current findings should help dispel concerns that affirmative hiring practices result in inferior women being hired over superior men (e.g., Niederle et al., 2013). Even though the Dr. Y foil was described as only slightly less accomplished, faculty almost always preferred to hire a slightly more accomplished candidate, and this preference was independent of the gender of the candidates and the gender of faculty raters, and it was observed in both math-intensive and non-intensive fields.

The absence of preference for male Dr. Y does not necessarily imply that academic hiring is meritocratic under all conditions. It is possible that with different levels of candidate information (or if the candidates involved were at a somewhat lower level as opposed to being in the top tier), different results might have been found. For example, in the Steinpreis et al. (1999) study no gender preferences were found when the candidate's CV was highly competitive, but a male preference was found when the CV was less strong. The current study is consistent with these results at the highly competitive candidate level, and showed that slightly less exceptional female candidates were not preferred over exceptional male candidates. Relatedly, Dovidio and Gaertner (2004) findings on aversive racism and selection decisions found that white participants did not discriminate against an unambiguously strong black candidate (vs. a white candidate), but discrimination occurred when the candidates' qualifications were depicted as ambiguous. These findings suggest that discrimination may be a concern when candidate qualifications are ambiguous, but not when candidates are exceptionally strong. Thus, the most prudent interpretation of the present results is that exceptionally strong candidates of both genders are unlikely to face gender discrimination. Given that the current study focused on top-tier candidates, any conclusions drawn should be confined to excellent tenure-track candidates.

The present findings may provoke concern of a different sort. If affirmative action is intended to not merely give a preference to hiring a woman over an identically-qualified man, but also to tilt the odds toward hiring a woman who may be slightly less accomplished but who is still rated very highly (recall that a 9.3 was in the “extremely impressive” range), gender diversity advocates may be disheartened by these findings. Those who have lobbied for more women to be hired in fields in which they are underrepresented, such as engineering and economics, may find the present findings dismaying and argue that, in the context of hiring in a field in which women are underrepresented, extremely well-qualified female candidates should be given preference over males rated a notch higher. Walton et al. (2013) argued on both empirical and theoretical grounds that hiring more members of devalued groups would actually promote meritocracy, diversity, and organizational performance, not undermine it. (Consideration of this argument entails complexities that are beyond the scope of this study.)

Notwithstanding differing views regarding affirmative hiring of impressive women in underrepresented fields, one claim finds no support in the present results. It is the allegation that the dearth of women in some fields is the result of superior women being bypassed in favor of less accomplished men—a claim made by numerous commentators.^3^If academic hiring is anti-meritocratic, then the weaker male Dr. Y should have been chosen over his stronger female competitors. But as seen, only 1.2% of males who were depicted as the slightly weaker candidate were preferred over slightly stronger female candidates. Thus, there is no support for the view that superior women are being bypassed in favor of inferior men when the contest is between highly accomplished candidates. Hence, these findings call into question claims of current biased tenure-track hiring that have been put forward and they suggest this is a propitious time for talented women to launch tenure-track careers in academic science, where their impressive credentials will be viewed favorably by hiring committees vis-à-vis identically-qualified men.

None of this means that women no longer face unique hurdles in navigating academic science careers. Evidence shows that female lecturers' teaching ability is down-rated due to their gender (Bug, 2010; MacNell et al., 2015), letter writers for applicants for faculty posts in chemistry and biochemistry use more standout (ability) words when referring to male applicants (Schmader et al., 2007), faculty harbor beliefs about the importance of innate brilliance in fields in which women's representation is lowest (Leslie et al., 2015), and newly-hired women in biomedical fields receive less than half the median start-up packages of their male colleagues, which could conceivably result in fewer publications down the line (Sege et al., 2015)—to mention a few areas where women continue to face hurdles. Nor do the present findings deny that historic sexist hiring prevented many deserving women from being hired. But these findings do call into question broad or unqualified claims of biased tenure-track hiring that have been put forward. The present findings are not incompatible with earlier studies that found anti-women bias at lower levels hiring a lab manager, (Moss-Racusin et al., 2012) or getting emails returned (Milkman et al., 2012), or hiring members of a math team (Reuben et al., 2014) if one assumes that bias may come into play when diagnostic information is missing (Koch et al., 2015) but not when such information is present as in the case of hiring a candidates who earned doctorates and garnered strong letters and ratings. This suggests that sex biases might reduce the number of women entering training for the STEM pipeline, but our results indicate that when a woman emerges as a strong candidate for a faculty position, she is no longer handicapped as far as being offered the job. Thus, these earlier findings of bias against less accomplished women (e.g., those applying to be lab managers) and the present findings are not mutually exclusive with the current results showing that top-tier female candidates are viewed favorably. This suggests that the gender gap in math-intensive fields might be best addressed by focusing on earlier experiences (encouraging more females to take high school AP physics, computer science, Calculus BC, recruiting more women into college STEM majors—areas identified by Ceci et al. (2014) as associated with the underrepresentation of women in these fields).

These new data will be of interest to academics struggling to increase the representation of women, because our data refute the claim that affirmative hiring policies are non-meritocratic and lead to less competent women being preferred for jobs. At the same time, these data debunk the claim that less-qualified men are favored over more-qualified women. We found no support for this, either. For those who believe affirmative action means giving a boost to an underrepresented group when all else is equal, our data will be welcome news, since we show that academic hiring preferences are quality-based. However, for those who argue that affirmative action means choosing slightly less accomplished individuals over more accomplished ones for reasons of diversity, our data suggest that at least when it comes to gender, faculty may be reluctant to embrace this pathway to diversity.

### Possible reactions to these findings

Our work on this topic has led to certain comments that we have heard repeatedly. We note some of these below along with a few reactions in response to them:

1. “*Thank goodness the academy is still a meritocracy in which competence determines who's hired*.” Some readers will likely be pleased that the academy is still a domain in which competence as traditionally quantified matters more than social factors. Such individuals believe that the academy should continue to function exactly this way, and are heartened when presented with evidence that it does.
2. “The 1–10 rating scale assumes that what is being rated is what matters—and women are often good at things not assessed by this scale, such as collaborative work, advising, and service.” Of course other attributes are important for a professorial career, attributes that are not measured by the scale we used which was based on research publications, teaching awards, job talk, and letters. However, the assumption of those who raise this point is that women are superior to men in these unmeasured skills—which is actually an empirical question. Men may be superior in them, or women may be, or both groups may be equivalent. Simply because an attribute is not assessed does not mean that women are superior at it, nor will inclusion of it necessarily boost women more than men, and close the gender gap (Ceci and Papierno, 2005).
3. “The entire male-centered, Western notion of assigning a job applicant a “quality” score on a 1–10 scale is misguided at its core.” Critics espousing this view are often in favor of a reinterpretation of everyday constructs. They eschew the notion that publications, awards, letters of reference, job talks, citations, and grants are the most important indicators of ability and predictors of success as a professor. Proposals for alternative, empirically-tested, valid and reliable indicators, and predictors of professorial success are most welcome, so that we can think more broadly.
4. “*Such ranking experiments have nothing to do with real-world hiring*.” As we noted, women have significant advantages in actual, real-world hiring—they are hired at higher rates than men. Some of our critics seem reluctant to acknowledge this fact, which is shown clearly in multiple audit studies that analyze who is actually hired at universities in the U.S. and Canada (see cites in Williams and Ceci, 2015). To argue that our experiment has no relevance to real-world hiring seems unpersuasive in view of the fact that in the real world of academic hiring women also are chosen over men in disproportionate numbers. As one commentator noted in arguing for the relevance of the current experimental design: “*One would have to say both that women are, in fact, stronger candidates (which is one strong assumption for which there is no direct evidence), implying that faculty don't prefer them over equally qualified men in real hiring contexts, and that, nonetheless, faculty DO prefer them in hypothetical situations (another strong assumption for which there is no direct evidence). By far the most sensible explanation is the most economical one: faculty prefer women both in the hypothetical case and the real case; their preferences don't swing wildly from the actual to the hypothetical.”*
5. “The process of assigning a rating to a woman's dossier is inherently prone to sexist bias; thus, women are less likely to receive an equivalent rating to that of male competitors.” This is a popular view; however, we found that subjects evaluating a single dossier, presented as either female or male, assigned a significantly HIGHER rating to that dossier when it belonged to a woman than a man (8.20 vs. 7.14, *p* < 0.01). The translation of traditional indicia (publications, letters, etc.) into ratings seems to work *at least as well* when ranking a female applicant.

### Limitations of present study

No experiment is perfect, and this one is no exception. It is possible that the faculty raters rarely chose the less competent candidate because they were supplied with “pre-ranked” quantitative ratings of the candidates (e.g., 9.3 or 9.5 on a 10-point scale). Hence, the present results may have been influenced by giving faculty “pre-ranked” ratings. Perhaps in the absence of being given quantitative ratings, faculty will shift criteria to justify their final decisions (e.g., be influenced by gender to give more credence to the eminence of an applicant's advisor/institution if a woman's list of publications is shorter than her male competitor's). Assigning pre-ranking scores will likely be variable in actual hiring; this variability in assigning scores could increase the rate of selecting someone who's rated a 9.3 on average. In other words, disagreements would likely be more common in actual hiring decisions due to the variable ways faculty translate their impressions. Since concerns about personality and teaching performance were raised for Dr. Y, but not for Drs. X or Y the primary reason someone might want to hire Dr. Y was gender when Y was a woman.

The present data provide no hint of the extent to which this occurs. However, in Experiment 5 of the Williams and Ceci (2015) paper, 127 faculty were given only one applicant to rank, either a man or woman who were identically accomplished. When the applicant was a man the faculty who were asked to rate his strength gave him a rating of 7.14 but when the identical portfolio belonged to a woman, the faculty who were asked to rate her gave her a rating of 8.2 (*p* < 0.01). So there is some suggestion that faculty shift their quantitative ratings to justify their preference for women, even when they are asked to generate the rating themselves, for what are actually identical accomplishments of both genders. If true, the present findings suggest this shift is limited to conditions in which candidates are identically competent and very accomplished.

In future research it would be interesting to vary the CVs of the 9.3-rated female applicant and the 9.5-rated male applicants in terms of their number of publications, advisor eminence, teaching awards during graduate school, the prestige of their PhD-granting institutions, etc. to determine how much shift in faculty-assigned quantitative ratings is observed as a function of applicant gender. In this experiment we began with the smallest difference of 9.5 vs. 9.3, with a plan to widen this gap if it turned out that faculty preferred slightly weaker women; but since they did not, there was no reason to widen the gap.

The low baserate for choosing the Y foil presents statistical issues: The rate of selecting the female Dr. Y (4.8%) was slightly higher than rate of selecting the male Dr. Y (1.2%), although this difference was not statistically reliable. Statisticians have written about the challenges of comparing frequencies of rare events (e.g., Bradburn et al., 2007). This has ramifications if the null result is affected by low statistical power, and future research might enlarge the sample size to see whether weaker women may be preferred over stronger men. However, faculty preference for the less qualified Dr. Y candidate was always rare in this experiment and in the Williams and Ceci (2015) one (<5%), regardless of applicant gender, so even if a preference for the weaker female became significant, the magnitude of such an effect would likely be quite small.

Although the current study is well-suited to address the specific question it posed, it employed a very specific methodology and DV that may have limited the operation and detection of implicit bias. It is possible that the use of implicit measures may have revealed bias as has been observed to occur even among university professors. Measures of explicit bias may not always be collinear with implicit measures (see Smyth and Nosek, 2015). As was noted in the introduction, findings from real-world hiring audits (not experiments, but actual hiring of university professors) indicate female applicants are typically hired at higher rates than their male counterparts—for at least the last two decades (Williams and Ceci, 2015).

Many have argued that the pro-women hiring preference is because women are on average stronger applicants, by dint of the winnowing process they have survived from college-to graduate school-to applying for tenure track jobs: it is argued that the reason women are more likely to be hired than their male counterparts for tenure track jobs in the real world is because those women who end up applying for tenure-track jobs represent the “cream of the cream,” a higher mean quality than the typical male applicant. Williams and Ceci (2015) designed their experiments to test this claim and reported that even when applicant strength was equated (experiments 1–3), faculty still preferred female applicants over identical male applicants. And as noted above, in their fifth experiment 127 faculty were asked to assign their own strength ratings (on a 10-point scale) to either a man or woman applicant. Faculty rated the same applicant 8.2 when it had a woman's name on it but only 7.14 when it had a man's name on it. So Dr. Y did not receive lower scores when described as a woman, and higher scores when described as a man, as some would predict.

Finally, the experimental condition that involved two female finalists (out of three) might have seemed odd for a STEM faculty member in math-intensive fields where 70%-plus of applicants are often male. On the flip side, having the woman be lower-rated than two men might have also made gender more salient. To the extent that either of these is true, it is an important issue that future research should address (e.g., by conducting focus groups or using a shortlists of only two applicants, only one of whom is female—a situation we deliberately rejected because we felt it might make the gender contest overly salient and explicit). However, in view of the media and publicity surrounding findings from these type of experimental designs, follow-up research cannot be undertaken in the near future without compromising the experimental reactivity of participants.

## Funding

Research was supported by NIH Grant 1R01NS069792-01.

### Conflict of interest statement

The authors declare that the research was conducted in the absence of any commercial or financial relationships that could be construed as a potential conflict of interest.

## Footnotes

^1^For example, a faculty respondent in the field of biology in Williams and Ceci's experiment wrote: “In a typical search these days we will receive over 200 applications for one position. The search committee triages that down to a group of around 30 or 40, and then no more than around 6–8 are invited to come for a three-day visit and to give a seminar.” Many similar comments were offered by others in their national survey, hence the finalists are usually unambiguously strong, as is true in our own department where a recent tenure-track search for an assistant professor generated 267 applicants in psychology. All applicants who survive to the short list are accomplished, having successfully completed doctorates, published papers, and garnered strong letters of recommendation. In a separate rating task we gave 35 faculty the CVs of actual short-listed candidates and asked them to rate these on a 10-point scale and, as expected, the mean rating was in the excellent range.

^2^With low counts some approximations used to compute CIs will not work well, so we used a number of methods to compute the CIs in R. The results were similar: The CIs for the ratio of choosing 7 Ys out of 158 pairings is between 2 and 9 percent, and the ratio of choosing 1 Y out of 92 pairings is between 0 and 6 percent. The CI of the difference in proportions covers 0 and ranges from −1.59 to 7.3 percent. Exact numbers below using the R code: (PropCIs) library(binGroup) binCI(158,7,.95) 95 percent CP confidence interval [ 0.018, 0.08915 ] Point estimate 0.0443 binCI(92,1,.95) 95 percent CP confidence interval [ 0.0002752, 0.05908 ] Point estimate 0.01087 scoreci(7,158,.95) data: 95 percent confidence interval: 0.0216 0.0886 scoreci(1,92,.95) data: 95 percent confidence interval: 0.0019 0.0590 binom.test(7,158) Exact binomial test data: 7 and 158 number of successes = 7, number of trials = 158, *p* < 2.2e^−16^ alternative hypothesis: true probability of success is not equal to 0.5 95 percent confidence interval: 0.01799534 0.08915030 sample estimates: probability of success 0.0443038 binom.test(1,92) Exact binomial test data: 1 and 92 number of successes = 1, number of trials = 92, *p* < 2.2*e*^−16^ alternative hypothesis: true probability of success is not equal to 0.5 95 percent confidence interval: 0.0002751557 0.0590778511 sample estimates: probability of success 0.01086957 add4ci(7,158,.95) data: 95 percent confidence interval: 0.02028254 0.09082857 sample estimates: [1] 0.05555556 add4ci(1,92,.95) data: 95 percent confidence interval: 0.06605514 sample estimates: [1] 0.03125 wald2ci(7,158,1,92,.95,adjust = T) data: 95 percent confidence interval: −0.01590209 0.07334890 sample estimates: [1] 0.0287234

^3^Many commentators have opined that female scientists are superior to their male counterparts, and therefore the fact that they are hired at the same rate as men obscures the fact that they should be hired at even higher rates, if merit was the basis for hiring. Consider:
“The studies [claiming gender neutrality] examined odds ratios rather than details of the proposals submitted. This does not rule out the possibility of gender bias. As Marie Vitulli and I said in 2011 [Kessel and Vitulli, 2011], “selection bias can also explain why, in the presence of gender discrimination, female scientists might still fare as well as their male colleagues in some respects if their work was better on average than that of their male peers.” (Kessel, 2012) “Given qualified women drop out of math-intensive fields at higher rates than their male peers. the women who remain are probably, on average, better than their male colleagues and should be having better (hiring) outcomes on average. If their salaries, resources, publication rates, etc. are similar, it then indicates gender discrimination still exists, not that this problem has been solved.” (http://blogs.sciencemag.org/sciencecareers/2011/02/the-real-cause.html; retrieved on June 22, 2014) “Female scientists were either not retained or not hired so that only a couple of super-brilliant female scientists were working in staff-scientist positions. On the other hand, several mediocre male scientists were hired and retained, many rising to staff-scientist positions or higher. If you compare these super-brilliant female scientists with their mediocre male counterparts, of course you will not see the difference in their treatment.” (Kali, 2011)
