# Supplementary material for: Women have substantial advantage in STEM faculty hiring, except when competing against more-accomplished men
Source: Front Psychol. 2015 Oct 20;6:1532. doi: 10.3389/fpsyg.2015.01532 (PMC4611984; doi:10.3389/fpsyg.2015.01532)
Supplement: Supplementary file 1 [file DataSheet1.DOCX]

**Appendix A**

A**ll emails, procedures, and protocols in this experiment conform to the relevant regulatory standards, and were approved by the Cornell IRB.** Two versions of the following email were sent to prospective faculty participants. The version below is the one in which the Y-foil was a woman. A mirror version was identical to this except that the gendered pronouns were flipped (every “he” became a “she”, every “her” became a “him”), so that all descriptions were counterbalanced across the genders of Drs. X, Y, and Z.

__________________________

**From:** Profs. Williams & Ceci
**Sent:** Monday, March 24, 2014 7:01 PM
**To:** Professor __________
**Subject:** Cornell Faculty-Hiring Study

_____________________________________________________________________________________

Dear Professor _________:

We have a quick favor to ask, one that will take less than 10 minutes. Our graduate students at Cornell are increasingly concerned about their ability to get tenure-track jobs in this weak economy. They repeatedly ask: "What type of graduate student is most likely to appeal to search committees, given that all top applicants have a solid research/publication record?" In an effort to help students throughout the natural and social sciences, we are collecting data on the types of applicants who are most hirable. We asked professors across the natural and social sciences to help us by providing descriptions of applicants for tenure-track jobs based on actual applicants they had interviewed recently, but with details specific to their discipline omitted. Now, we are asking colleagues such as you to help by rating three hypothetical short-listed job candidates. The results, once your name and all identifying information is removed, will be posted on a website, where hopefully they will be useful to graduate students and their advisors across fields. Cornell's IRB requires us to remind you that the internet is neither private nor secure, and that you are obviously under no obligation to help; and also to state that all personal information in your response (name, email, institution) will be destroyed. Research like this would not be possible without the generous help of fellow scientists such as you. We are very grateful for your participation and wish you and your students the best.

Sincerely,

Steve Ceci & Wendy M. Williams

Professors, Cornell University

**DESCRIPTIONS OF 3 CANDIDATES (X, Y, AND Z)**

Imagine you are on your department's personnel/search committee. Your department plans to hire one person at the entry assistant-professor level. Your committee has struggled to narrow the applicant pool to three short-listed candidates (below), each of whom works in a hot area with an eminent advisor. The search committee evaluated each candidate's research record, and the entire faculty rated each candidate's job talk and interview. Now it is your turn to rank the candidates in order of hiring preference. Please read the search committee chair's notes below and rate each candidate.

**Dr. X:** X struck the search committee as a real powerhouse. Based on his vita, letters of recommendation, and their own reading of his work, the committee rated X's research record as "extremely strong." X's recommenders all especially noted his high productivity, impressive analytical ability, independence, ambition, and competitive skills, with comments like "X produces high-quality research and always stands up under pressure, often working on multiple projects at a time." They described his tendency to "tirelessly and single-mindedly work long hours on research, as though he is on a mission to build an impressive portfolio of work." He also won a dissertation award in his final year of graduate school. X's faculty job talk/interview score was 9.5/10. At dinner with the committee, he impressed everyone as being a confident and professional individual with a great deal to offer the department. During our private meeting, X was enthusiastic about our department, and there did not appear to be any obstacles if we decided to offer him the job. He said our department has all the resources needed for his research.

**Dr. Y:** Y came across during her interview/visit as a smart, serious scholar with a solid record. Based on her vita, letters of recommendation, and their own reading of her work, the committee rated her research record as "very strong." Y's letter-writers all praised the "breadth and quality of her research and ideas" and described her as a "highly desirable hire among her cohort of graduate students." They also noted that Y works on an "established set of paradigms sure to continue to generate publications and funding in the future." Y's faculty job talk/interview score was 9.3/10. One issue raised by two members of the search committee is that Y is somewhat shy and reserved; thus there was some question about her ability to handle large introductory lecture courses. No one foresaw any problems with her teaching in a small-seminar context. At dinner, Y was pleasant but spoke little and was a bit hard to get to know—however, most of us felt this would resolve in time. During my private discussion with Y, she seemed enthusiastic about our department, and there did not appear to be any obstacles if we decided to offer her the job. She said our department has all the resources she needs for her research.

**Dr. Z:** Z impressed the entire search committee as a great potential hire. Based on his vita, letters of recommendation, and their own reading of his work, the search committee rated Z's research record as "extremely strong." Letter-writers especially noted that Z is highly creative and original in his approach to scholarship, with comments like "Z is poised to break new ground with his unique and imaginative applications of his advisor's theory, and is sure to change how people think about his research area." They also described Z's impressive teaching abilities, mentioning that he was "widely considered an effective and supportive mentor by the junior graduate students and undergraduates he worked with." He also won a teaching award in graduate school. Z's faculty job talk/interview score was 9.5/10. At dinner with the committee, he reached out to everyone, showing himself to be very likeable, kind, and socially skilled. During our private meeting, Z was enthusiastic about our department, and there did not appear to be any obstacles if we decided to offer him the job. He said our department has all the resources needed for his research.

Please rate each applicant using the following scale, ranging from 1 to 10:

10=truly extraordinary/exceptional

9=extremely impressive

8=high-excellent

7=low-excellent

6=extremely good

5=very good

4=good

3=acceptable

2=marginally acceptable

1=cannot support

Please rate each candidate:

X_____

Y_____

Z_____

Please rank each candidate:

#1 (best)_____

#2 (2^nd^ best)_____

#3 (3^rd^ best)_____

Your position:

Assistant_____

Associate_____

Full_____

Year of Ph.D. _______

Major Field/Discipline____________________

Approximately how many times have you served on a search committee to hire at the assistant professor rank?_____

Optional comments?

Check here if you want the url for the website once data are available._____

THANK YOU!

For example, a faculty respondent in the field of biology in Williams & Ceci’s experiment wrote: "In a typical search these days we will receive over 200 applications for one position. The search committee triages that down to a group of around 30 or 40, and then no more than around 6 to 8 are invited to come for a three-day visit and to give a seminar." Many similar comments were offered by others in their national survey, hence the finalists are usually unambiguously strong, as is true in our own department where a recent tenure-track search for an assistant professor generated 267 applicants in psychology. All applicants who survive to the short list are accomplished, having successfully completed doctorates, published papers, and garnered strong letters of recommendation. In a separate rating task we gave 35 faculty the CVs of actual short-listed candidates and asked them to rate these on a 10-point scale and, as expected, the mean rating was in the excellent range.

With low counts some approximations used to compute CIs will not work well, so we used a number of methods to compute the CIs in R. The results were similar: The CIs for the ratio of choosing 7 Ys out of 158 pairings is between 2 and 9 percent, and the ratio of choosing 1 Y out of 92 pairings is between 0 and 6 percent. The CI of the difference in proportions covers 0 and ranges from -1.59 to 7.3 percent. Exact numbers below using the R code:

(PropCIs)
library(binGroup)

binCI(158,7,.95)

95 percent CP confidence interval
[ 0.018, 0.08915 ]
Point estimate 0.0443
binCI(92,1,.95)

95 percent CP confidence interval
[ 0.0002752, 0.05908 ]
Point estimate 0.01087

scoreci(7,158,.95)

data:

95 percent confidence interval:
0.0216 0.0886

scoreci(1,92,.95)

data:

95 percent confidence interval:
0.0019 0.0590

binom.test(7,158)

Exact binomial test

data: 7 and 158
number of successes = 7, number of trials = 158, p-value < 2.2e-16
alternative hypothesis: true probability of success is not equal to 0.5
95 percent confidence interval:
0.01799534 0.08915030
sample estimates:
probability of success
0.0443038

binom.test(1,92)

Exact binomial test

data: 1 and 92
number of successes = 1, number of trials = 92, p-value < 2.2e-16
alternative hypothesis: true probability of success is not equal to 0.5
95 percent confidence interval:
0.0002751557 0.0590778511
sample estimates:
probability of success
0.01086957

add4ci(7,158,.95)

data:

95 percent confidence interval:
0.02028254 0.09082857
sample estimates:
[1] 0.05555556

add4ci(1,92,.95)

data:

95 percent confidence interval:
0.00000000 0.06605514
sample estimates:
[1] 0.03125

wald2ci(7,158,1,92,.95,adjust = T)

data:

95 percent confidence interval:
-0.01590209 0.07334890
sample estimates:
[1] 0.0287234

Many commentators have opined that female scientists are superior to their male counterparts, and therefore the fact that they are hired at the same rate as men obscures the fact that they should be hired at even higher rates, if merit was the basis for hiring. Consider:

*“The studies [claiming gender neutrality] examined odds ratios rather than details of the proposals submitted. This does not rule out the possibility of gender bias. As Marie Vitulli and I said in 2011 [Kessel & Vitulli, 2011], “selection bias can also explain why, in the presence of gender discrimination, female scientists might still fare as well as their male colleagues in some respects if their work was better on average than that of their male peers.” (Kessel, 2012)*

*“Given qualified women drop out of math-intensive fields at higher rates than their male peers . . . the women who remain are probably, on average, better than their male colleagues and should be having better (hiring) outcomes on average. If their salaries, resources, publication rates, etc. are similar, it then indicates gender discrimination still exists, not that this problem has been solved.” (*[*http://blogs*](http://blogs)*. sciencemag.org/sciencecareers/2011/02/the-real-cause.html; retrieved on June 22, 2014)*

*“Female scientists were either not retained or not hired so that only a couple of super-brilliant female scientists were working in staff-scientist positions. On the other hand, several mediocre male scientists were hired and retained, many rising to staff-scientist positions or higher. If you compare these super-brilliant female scientists with their mediocre male counterparts, of course you will not see the difference in their treatment.” (Kali, 2011)*
